# 

**Published:** 1880-04

**Authors:** 


### CONTENTS FOR APRIL, 1880.

ORIGINAL COMMUNICATIONS.

PAGE.

ART. I.—Treatment of Fracture of the Jaw, with Critical Remarks. By THOMAS BRIAN GUNNING, D. D. S. .......... 171

ART. II.—Observations on Dr. Taylor's Paper on Flagellation of the Abdomen, Etc. By RUFUS W. GRISWOLD, M. D. .......... 185

ART. III.—Ideal Dentistry. By C. T. STOCKWELL. 191

ART. IV.—Acute Rheumatism in a Child. By BARTON DOZIER, M. D. .......... 194

Meeting of the Georgia Medical Society, of Savannah .......... 196

ECLECTIC DEPARTMENT.

The Abuses of Medical Charity .......... 198

Tooth-Caries of Pregnancy .......... 200

EDITORIAL DEPARTMENT.

Specialties and Specialists .......... 204

Hospitals, their Location, etc. .......... 205

Asphyxia in the Drowned, etc. .......... 207

Some of our Medical Universities and Colleges... 208

Proceedings of the Georgia Medical Society...... 209

Meetings of Medical and Dental Societies....... 215

To our Friends and Correspondents.......... 209

BIBLIOGRAPHICAL DEPARTMENT.

Headaches: their Nature and Treatment........ 210

Yellow Fever: a Nautical Disease.......... 210

Transactions of the American Med. Association... 212

Periodicals, Pamphlets, etc. .......... 213

Tit for Tat .......... 216

HYMENEAL. NATALITIAL. NECROLOGICAL....... 217

Mortuary Report for March .......... 218

Meteorological " " .......... 219

Special Notice .......... 220

